# Do Patterns of Bacterial Diversity along Salinity Gradients Differ from Those Observed for Macroorganisms?

**DOI:** 10.1371/journal.pone.0027597

**Published:** 2011-11-18

**Authors:** Jianjun Wang, Dongmei Yang, Yong Zhang, Ji Shen, Christopher van der Gast, Martin W. Hahn, Qinglong Wu

**Affiliations:** 1 State Key Laboratory of Lake Science and Environment, Nanjing Institute of Geography and Limnology, Chinese Academy of Sciences, Nanjing, China; 2 Life Sciences College, Anhui Normal University, Wuhu, China; 3 National Environment Research Council (NERC) Centre for Ecology and Hydrology, Wallingford, United Kingdom; 4 Institute for Limnology, Austrian Academy of Sciences, Mondsee, Austria; Argonne National Laboratory, United States of America

## Abstract

It is widely accepted that biodiversity is lower in more extreme environments. In this study, we sought to determine whether this trend, well documented for macroorganisms, also holds at the microbial level for bacteria. We used denaturing gradient gel electrophoresis (DGGE) with phylum-specific primers to quantify the taxon richness (i.e., the DGGE band numbers) of the bacterioplankton communities of 32 pristine Tibetan lakes that represent a broad salinity range (freshwater to hypersaline). For the lakes investigated, salinity was found to be the environmental variable with the strongest influence on the bacterial community composition. We found that the bacterial taxon richness in freshwater habitats increased with increasing salinity up to a value of 1‰. In saline systems (systems with >1‰ salinity), the expected decrease of taxon richness along a gradient of further increasing salinity was not observed. These patterns were consistently observed for two sets of samples taken in two different years. A comparison of 16S rRNA gene clone libraries revealed that the bacterial community of the lake with the highest salinity was characterized by a higher recent accelerated diversification than the community of a freshwater lake, whereas the phylogenetic diversity in the hypersaline lake was lower than that in the freshwater lake. These results suggest that different evolutionary forces may act on bacterial populations in freshwater and hypersaline lakes on the Tibetan Plateau, potentially resulting in different community structures and diversity patterns.

## Introduction

A widely held ecological tenet states that a more extreme environment is expected to maintain lower species diversity [1]. From the physiological point of view of an organism, an extreme condition is defined by levels of environmental factors, the effects of which pose difficulties for the functioning of the organism [2], [3]. From the ecological point of view of a community, extreme conditions can be defined by levels of environmental factors, the effects of which pose difficulties for the survival of specific taxa or all taxa. In both cases, a given factor can range from normal to increasingly extreme levels and can create a different community composition and diversity. A typical example of such an environmental factor is salinity, which varies across natural aquatic systems due to differences in the ratio of precipitation to evaporation or the input of dissolved ions from the catchment. In inland waters, salinity ranges from <1‰ (freshwater systems) to approximately 400‰ (e.g., Don Juan Pond, Wright Valley, Antarctica), and many lakes are characterized by salinities greater than the salinity of ocean water (∼35‰). A decreasing trend in floral and faunal species richness with increasing salinity in inland waters in generally observed (e.g. [4], [5]). However, this pattern seems to be less reproducible if applied to microbial communities (e.g. [6], [7], [8]), although microbial ecologists found several lines of evidence for a decreasing trend of microbial diversity along gradients of increasing salinity [9], [10]. Even in habitats that are traditionally considered extreme (i.e., high-salinity habitats), the overall microbial communities were still surprisingly diverse (e.g. [11]). Given that the dominant halophiles are from microbial taxa [2], it is reasonable to assume that the pattern of microbial species richness along salinity gradients may deviate from the general principle, especially when the ‘extreme’ concept is viewed from the perspective of halophiles or a halophilic community.

Traditionally, microbial research in saline environments has been conducted for the following reasons: (i) to help search for potential biotechnological applications; (ii) to study potential forms of extinct and/or extant life on Mars; and (iii) to understand the early evolution of the biosphere on Earth (Reviewed in [12]). Lozupone and Knight [13] recently postulated that microbial research in saline and related environments could contribute to the study of the broad-scale distribution of microbial communities and help to discern the potential factors underlying global microbial diversity patterns. For example, it has been shown that salinity is the primary factor regulating the global diversity patterns of bacteria [13], [14], archaea [15] and the functional gene lineages involved in the denitrification pathway [16] or the hydrolysis of chitin [17]. Thus, it is expected that microbial studies along salinity gradients could shed additional light on this new point.

The majority of studies conducted to investigate the influence of salinity on the distribution of microbial diversity have focused on temporally dynamic saline systems, such as estuaries [18], [19], [20] and coastal solar salterns [21], whereas fewer investigations have focused on stable and natural saline lakes (Table S1), which have evolved over long periods of time from freshwater lakes [22], [23]. The Tibetan Plateau comprises several thousand lakes, covering a total area of 4.5×10^4^ km^2^. Previous studies have demonstrated the presence of an extensive geographic salinity gradient among the Tibetan lakes and have shown that salinity strongly controls the community composition of bacterioplankton [22]. This study utilized the bacterioplankton of Tibetan lakes as a model community and salinity as the dominant environmental factor. The objectives of this study were as follows: (1) to ascertain whether the bacterioplankton richness along the salinity gradient follows the general tenet, i.e., a decrease with increase in salinity and (2) to reveal the potential causes of the observed taxon richness patterns along salinity gradients.

## Materials and Methods

### Selection and sampling of lakes

During June and July of 2005, 24 lakes located in the eastern part of the Tibetan Plateau at elevations ranging from 2,790 to 4,619 m above the sea level were sampled (Table S2). No specific permits were required for sampling these lakes or for the described field studies. Because most of the sampled lakes are polymictic, we took one sample from the deepest region of each lake. Nine water samples collected 50 cm from the surface of 9 Tibetan lakes during 2004 [24] were also included in this study. The geographical range of the 2005 samples (34.10°–37.83°N, 95.08°–100.74°E) was smaller than that of the 2004 samples [24]. The elevation range of the 2005 samples was also smaller than that of the 2004 samples (2,817–5,134 m). However, the salinity range of the 2005 samples (0.3–279.2‰) was greater than that of the 2004 samples (0.2–222.6‰) [24]. This sampling scheme minimized the effects of geographic factors and strengthened the effects of environmental factors because geographic variables, such as altitude, can act to structure the composition of the bacterioplankton community [22]. For detailed descriptions of the sampled lakes, see Table S2.

The sampling procedures used in this study have been described elsewhere [22], [24]. In brief, water samples were collected from the surface waters (the top 50 cm) with a 5-L Schindler sampler because previous work indicated that minor variations of the community composition of the bacterioplankton occurred along the depth profile due to daily water mixing [22], [24]. Water samples (50 mL) were preserved with 2% formaldehyde (final concentration) on-ite and stored at 4°C in the dark for the subsequent quantification of bacteria (analyzed within 2 months). The plankton samples (250–500 mL water) for the denaturing gradient gel electrophoresis (DGGE) analyses were collected on 0.2-µm pore size Isopore filters using a hand pump at a pressure less than 15 mm Hg. Filters for the extraction of DNA were stored in liquid nitrogen until arrival in the laboratory. Untreated water samples of 2–3 L were transported to the laboratory for immediate chemical analysis. The water temperature, pH, conductivity, and Secchi-depth were measured on-site. The concentrations of the eight major ions potassium (K^+^), sodium (Na^+^), calcium (Ca^2+^), magnesium (Mg^2+^), chloride (Cl^−^), sulphate (SO_4_^2−^), carbonate (CO_3_^2−^), and bicarbonate (HCO_3_^−^), and the concentrations of total nitrogen (TN) and total phosphorus (TP) were measured according to standard methods [25]. The salinity (salt concentration) of the investigated habitats was determined by the sum of the concentrations of the eight major ions [26].

### Microbial counts, DNA extraction and purification

Total bacterial numbers were determined by epifluorescence microscopy after DAPI (4′,6-diamidino-2-phenylindole) staining as described previously [27]. DNA was extracted from the biomass collected on the filters by phenol-chloroform extraction, precipitated with ethanol, purified using a Wizard DNA Cleanup kit (Promega, Shanghai, China), and subsequently concentrated to a volume of 50 µL [22].

### Polymerase chain reactions (PCR) and DGGE

We investigated the bacterial community composition by DGGE with amplicons of partial 16S rRNA genes amplified with nested group-specific PCR. Several previous studies have demonstrated that using the nested group-specific amplification of ribosomal markers improves the resolution of the phylogenetic fingerprinting of bacterial communities [28], [29]. In the first PCR step, nearly complete 16S rRNA genes of all of the bacteria were amplified using the bacterial primers 8f and 1492r [30]. In the second PCR step, aliquots of the PCR products obtained in the first step were used for a re-amplification with group-specific primer sets for *Actinobacteria*, *Bacteroidetes*, *Cyanobacteria*, *Firmicutes*, *Planctomyces*, *Alphaproteobacteria* and *Betaproteobacteria*. The group-specific primers were 517f/AB1165r, CFB319f/907r, CYA359f/CYA781r-a (CYA781r-b), LGC354f/907r, 8f/PLA886r, 517f/Alf968r, and Beta680f/1055r, respectively (Table S3). The PCR conditions are listed in Table S3. In addition to the group-specific DGGE analysis, an analysis of the total bacterial communities was performed using the primers 341f (with a 40-bp GC clamp) and 907r [31]. The DGGE was performed and analyzed using the methods described in our previous report [32]. The DGGE band number was used as a proxy of bacterial taxon richness [32].

### Cloning, sequencing, and phylogenetic analyses of 16S rRNA genes

Kelike Lake and Chaqia Lake, representing low (0.71‰) and high salinities (279.2‰) (Table S2), respectively, were selected to construct clone libraries for a more detailed analysis of their bacterial community structure. Aliquots of the products from the PCRs and re-PCRs of the two lakes were ligated into the pMD18-T vector (Takara, Dalian, China) and transformed into TOP10 competent cells. Further details of the clone library construction can be found in our previous report [32]. For each primer pair, 72 clones from each environment were randomly selected for sequencing. The insert fragments resulting from the 8f/1492r, 8f/PLA886r and AC517f/AC1165r primer pairs were sequenced with the primer 8f, 8f and AC517f, respectively (Table S3). Other fragments were sequenced with the M13f primer (5′ -GTAAAACGACGGCCAGT- 3′).

The clone sequences obtained were manually checked for chimeras using the Ribosomal Database Project II (http://wdcm.nig.ac.jp/RDP) prior to submission to GenBank (http://www.ncbi.nlm.nih.gov/genbank). Furthermore, the submitted sequences were analyzed in the context of the complete data set to identify putative anomalies using Bellerophon [33] and Mallard [34] with the default settings. Fifty putative chimeric sequences were identified and these were excluded from the subsequent analyses and retained in GenBank with an organism name of ‘uncultured bacterium’ and a note stating ‘putative inter-phylum chimera’.

Sequences were initially aligned with the NAST algorithm from the GreenGenes database [35]. Maximum likelihood trees were constructed by using the Dnaml program in the Phylip package [36]. The rate of diversification within each environment was examined using the tree shape statistic, γ [37], [38], which is extraordinarily sensitive to recent diversification rates [39]. We transformed the maximum likelihood trees to ultrametric trees (rooted trees with edge lengths where all of the leaves are equidistant from the root) by using non-parametric rate smoothing [40]. Under the pure-birth model, the gamma statistic equals zero. Negative values of gamma indicate that the phylogeny's internal nodes are closer to the root than expected under the pure-birth model and imply a deceleration in the accumulation of lineages, whereas positive values indicate that the phylogeny's internal nodes are closer to the tips and imply an acceleration of the accumulation of lineages [37]. To provide a global picture of diversification rates, the gamma statistics were calculated for the total sequences and the sequences obtained from the 8f/1492r primer for each of the two lakes. All of the analyses were conducted in the R environment (http://www.r-project.org) with the packages geiger v1.2-14 and ape v2.3-1.

### Statistical analyses

Two types of data matrices were constructed from the 2005 samples. The matrices of the data on the bacterial taxa were generated from the presence and absence of DGGE bands. The environmental matrices included 16 environmental variables (shown in part in Table S2). All of the environmental variables, except the pH, were log-transformed. The band numbers of the 341f/907r DGGE fingerprints and the sum of the bands from the phylum-specific-primer DGGE fingerprints were used as proxies for the bacterial taxon richness. Principal component analysis (PCA) was performed on the latter environmental matrix to determine the primary environmental gradient. To investigate the relationship between the environmental factors and the bacterial communities, a canonical correspondence analysis (CCA) was performed using the two matrices. The significance of the relationship between the environmental factors and the BCC was tested with a Monte Carlo permutation tests (499 permutations). A nonparametric Spearman correlation was applied to analyze the DGGE band numbers and environmental factors for all of the samples from 2005 along the entire salinity range and low salinity range (salinities <1‰), respectively. Richness trends along the investigated salinity gradient were analyzed using a locally weighted scatterplot smoothing regression (LOWESS regression, span 2/3; degree 1) to detect major nonlinearities or a generalized linear model to detect linearity. Furthermore, we calculated both the phylogenetic diversity and the community taxon richness with maximum likelihood trees from clone sequences under different sequence-similarity cutoffs (95%, 97%, 99%, and 100%). The phylogenetic diversity was described by Faith's PD as the total length of the phylogenetic branches connecting the species within a community [41]. The taxon richness was obtained with a non-parametric estimator: Chao1 = S_obs_+(a^2^/[2*b]), where *S_obs_* is the number of species observed and *a* and *b* are the number of species observed exactly once and twice, respectively [42]. We then calculated the taxon richness or phylogenetic diversity with a rarefaction analysis (1,000 times). The rarefaction curves were plotted against the sequences sampled to compare the phylogenetic or community diversity under different sequence-similarity cutoffs. CCA analysis was performed with the software Canoco v4.53, and other statistical analyses were conducted in the R environment (http://www.r-project.org) with the package Vegan v1.16-2.

### Nucleotide sequence accession numbers

The partial 16S rRNA gene sequences were deposited in GenBank under the accession numbers FJ843650–FJ844359.

## Results

### Physico-chemistry and bacterioplankton community composition of the lakes

The major geographical and physicochemical characteristics of the 24 lakes investigated in 2005 are summarized in Table S2 The characteristics of the lakes sampled in 2004 are given in our previous study [22]. The entire set of samples covered a salinity range from 0.2 to 279.2‰. The PCA revealed that the first component explained 80.5% of the variation in the physicochemical conditions of the lakes investigated. Salinity was the dominant factor that explained this component (Fig. S1).

**Figure 1:**
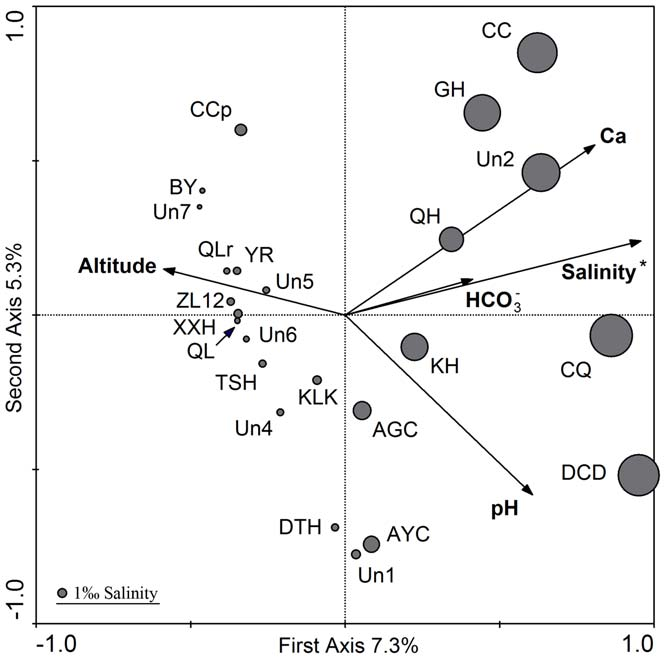
Canonical correspondence analysis based DGGE data and environmental factors in 2005. The plot shows that the differentiation of bacterioplankton communities was significantly related to salinity (*P*<0.01 with 499 permutations, Monte Carlo permutation tests). The scale of circles (provided in the lower-left corner) represents salinity concentration. More details on the abbreviations of the lakes, see Table S1.

We first included all of the environmental variables into the CCA model used for explaining bacterioplankton community composition. Among the explanatory variables, salinity was the most important factor for species composition. To reduce the collinearities among the explanatory variables, the variables with variance inflation factors greater than 20 were dropped sequentially from the model [43]. Five variables were retained in the final model in which only salinity was significant (*P*<0.01) (Fig. 1).

### Bacterioplankton abundance and taxon richness

**Figure 2:**
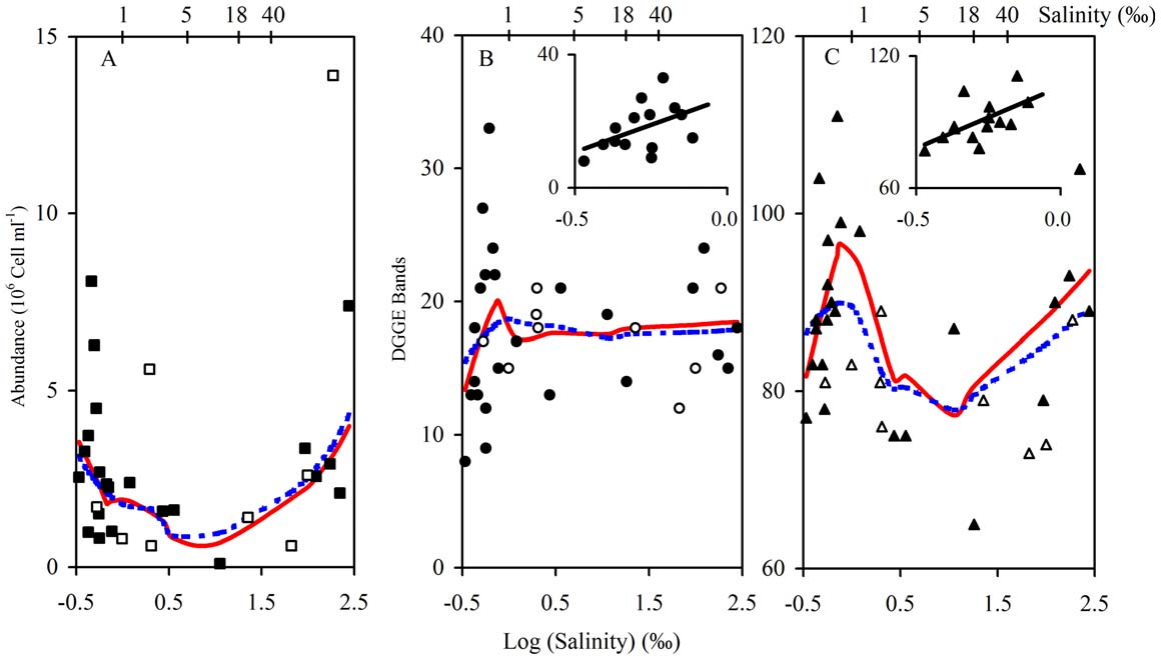
Trends of prokaryotic abundance and bacteria taxon richness (represented by the number of DGGE bands) along the salinity gradient (▪, •, ▴: 2005 samples; □, ○, ▵, 2004 samples). A) Prokaryotic abundance, solid and dotted lines represented the trends for 2005 samples and the whole set of samples (including samples from 2004), respectively, which were analyzed using LOWESS regression (span 2/3; degree 1; the same in B and C). B) DGGE bands obtained with the 8f - 1492r primers. C) Sum of DGGE bands obtained with seven phylum-specific primers. The small graphs in B and C show exclusively the data for the salinity range 0–1‰ (freshwater samples) collected in 2005 including a linear regression line obtained by using a generalized linear model (R^2^ = 0.21, *P* = 0.094 and R^2^ = 0.35, *P* = 0.028 respectively).

The total prokaryotic abundance of the 2005 samples varied by more than one order of magnitude, from 1.0×10^5^ to 8.1×10^6^ cells ml^−1^, and did not show a systematic change along the entire salinity gradient (Fig. 2A). This pattern remained nearly unchanged when the samples collected in 2004 were included (Fig. 2A).

The number of DGGE bands obtained with the bacterial primers ranged from 8 to 33 for the 2005 samples and did not show a decreasing pattern along the salinity gradient (Fig. 2B). This finding was consistent with the results for the samples collected in 2004. A band-number peak occurred at salinities of approximately 1‰ (i.e., at the transition from freshwater to oligosaline waters). The number of DGGE bands increased with salinity in the range of up to approximately 1‰, but no obvious further increase or decrease of band numbers with salinity was observed for salinities of >1‰ (Fig. 2B).

The summed DGGE band numbers obtained with the seven phylum-specific primer pairs varied between 65 and 111 for the 2005 samples and showed no decreasing trend along the salinity gradient (Fig. 2C). A band-number peak occurred at salinities of approximately 1‰ and the band number increased with salinities up to approximately 1‰ (Fig. 2C). As was the case for the DGGE band numbers obtained with the general bacterial primers, the general pattern of the summed band numbers did not change when the samples collected in 2004 were included (Fig. 2C). To test if the salinity pattern of the summed DGGE bands of the seven phylum primers was a chance occurrence, we randomly summed the bands obtained from 4, 5 or 6 primer pairs and then used LOWESS regression to visualize the patterns (Fig. S2). Interestingly, similar patterns related to salinity were observed for almost all of the random combinations of these primer pairs. Only a few exceptions were noted: for example, the pattern obtained without the primer pairs of *Cyanobacteria*, *Firmicutes* and *Planctomyces*.

Spearman's correlation indicated that, over a low salinity range (salinity <1‰), the DGGE band numbers for all of the 2005 samples (estimated either from the bacterial primers or from the summed DGGE bands) were positively correlated with the salinity and the concentration of magnesium, whereas no such significant relationship was found over the entire salinity range (Table S4). The concentration of total nitrogen was also found to be marginally significantly correlated with the DGGE numbers over a low salinity range when the summed bands of the phylum-specific-primer DGGE fingerprints were used (*P* = 0.05) (Table S4).

### Clone library phylogenetic analyses

**Figure 3:**
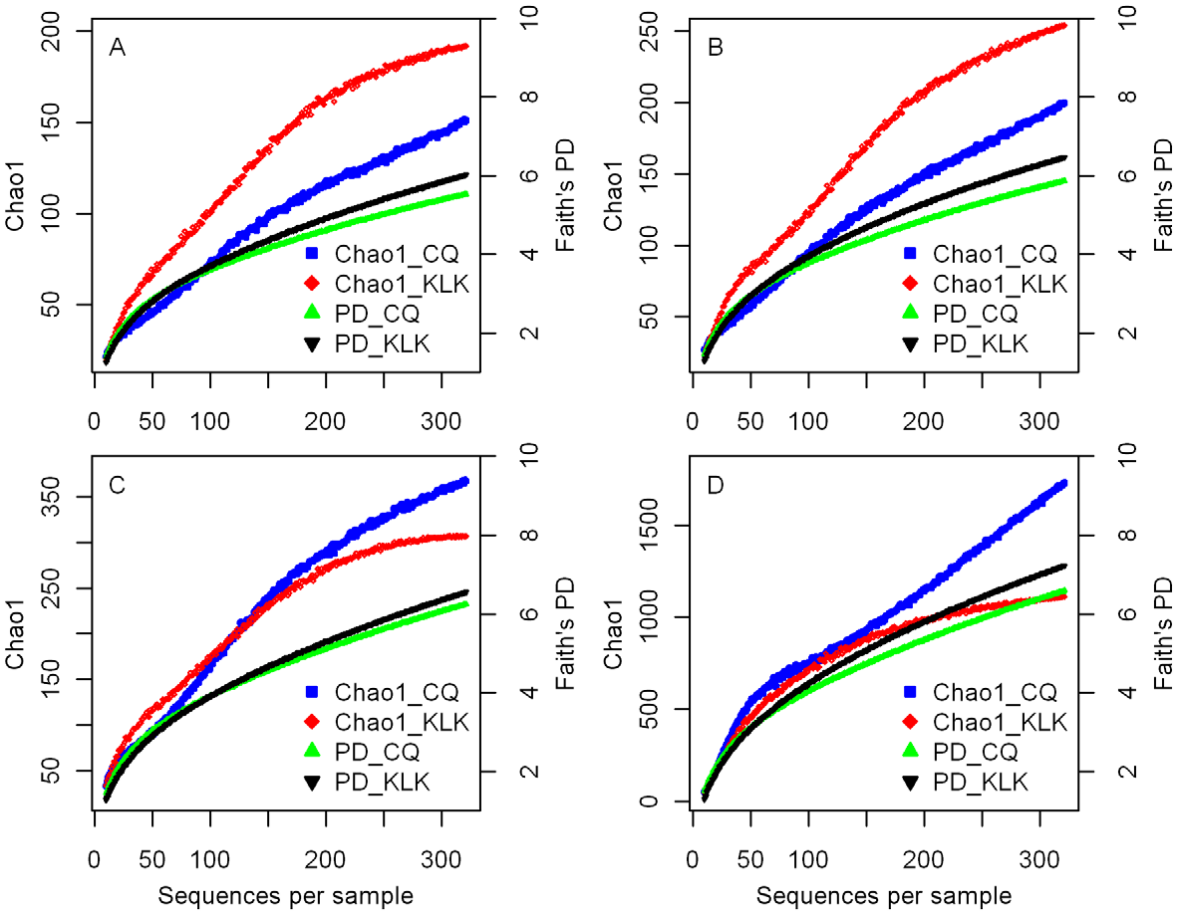
Rarefaction plots indicating community taxon richness (Chao1) and phylogenetic diversity (Faith's PD) based on 16S rRNA gene sequences obtained from Lake Kelike (KLK, low salinity) and Lake Chaqia (CQ, high salinity). Plots A–D depict results obtained with different similarity cutoffs (A: 95% cutoff; B: 97% cutoff; C: 99% cutoff; D: no cutoff value).

In total 660 bacterial sequences (339 for Chaqia Lake and 321 for Kelike Lake) retrieved with the bacterial and phylum-specific primers were analyzed. The gamma statistics calculated for the total sequences recovered with all of the primers and those using the bacterial primers were both significantly higher for Chaqia Lake than for Kelike Lake and were all greater than zero (Table 1). The rarefaction plots of Faith's PD showed that the total length of the phylogenetic branches connecting the species within the community in Kelike Lake was higher than the corresponding value in Chaqia Lake under different sequence-similarity cutoffs (Fig. 3A, B, C, D). In contrast, less-consistent patterns for community taxon richness (measured by Chao1) were found when different similarity cutoffs were applied. Chao1 for Kelike Lake was higher than that of Chaqia Lake under both 95% and 97% similarity cutoffs (Figs. 3A, 3B), whereas it was lower than that of Chaqia Lake under higher similarity cutoffs (99% and 100%) (Figs. 3C, 3D).

**Table 1:** Tree shape statistics (gamma) of the community phylogenies from Chaqia and Kelike lakes, based on maximum-likelihood phylogenetic analyses.

	Chaqia Lake		Kelike Lake	
	Clones	Gamma	Clones	Gamma
Bacteria 8f	38	2.64±0.69	35	1.88±0.84
Bacteria Sum	339	19.00±0.52	321	18.48±0.53

8f: clone sequences obtained with 8f/1492r; Sum: clone sequences obtained with 8f/1492r and phylum-specific primers (More details on the primer pairs, see Table S2). The numbers of sequences randomly sampled for calculating gamma statistics (10,000 times) were 30 and 300 for 8f and Sum, respectively. There were significant differences in the generated gamma statistics between Chaqia and Kelike Lake both for 8f and Sum (independent 2-group t-test, *P*<0.001).

## Discussion

Understanding species richness patterns along environmental gradients has long been of major interest to ecologists. However, the influence of such gradients on microbial taxon richness has remained relatively less studied (e.g. [44]). To investigate the microbial diversity pattern along such gradients, we used salinity as an environmental factor and selected bacterioplankton as a model community. Our results confirmed our previous findings [22] that salinity was among the significant factors that control the bacterioplankton community at the regional scale, although we cannot exclude other factors, such as biotic interactions, seasonal variations or geographical variables. These results are consistent with the hypothesis that salinity is a dominant environmental filtering force for prokaryotic community composition at the global scale [13], [14], [15], [16].

**Figure 4:**
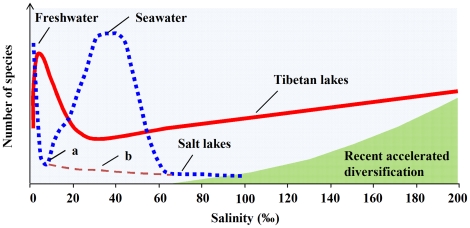
Conceptual model depicting differences in richness patterns between prokaryotes (solid curve) and macroorganisms (dotted curve, mainly modified from Purves et al. [**49**] and references therein (e.g. [**50**])) along salinity gradients. The shaded area indicates the increasing influence of recent accelerated diversification on the taxon richness of bacteria. a, brackish water; b, saline inland water, which was summarized from a review [51] (and a reference therein [52]) and published references of different functional groups (i.e., macrophytes [5], macroinvertebrates [4]).

A minimum requirement for any data set to reveal biological diversity change over an environmental gradient is that it includes data spanning the entire gradient over which changes in diversity patterns are expected to occur [45]. However, such ‘complete’ gradients with sufficient natural samples have been infrequently accessed by previous studies on bacterial diversity along salinity gradients (Table S1). We thus sampled 32 Tibetan pristine lakes with a salinity range from 0.2‰ to 280‰ to approach to a truly “complete” salinity gradient. This relatively large number of samples along a broad salinity range in natural lakes allows for the observed bacterial richness patterns to be subjected to appropriate statistical analyses and hypothesis testing. In contrast to the estimation of the species diversity of macroorganisms using morphological analyses, different molecular methods were used to determine microbial diversity in previous studies, which including DGGE, t-RFLP (terminal-restriction fragments length polymorphism) and ARISA (amplified ribosomal intergenic spacer analysis) (Table S1). In this study, DGGE was applied for the bacterioplankton community analysis, and the DGGE band number was used as a proxy for the bacterial taxon richness. Because DGGE may only estimate the number of taxa for the most abundant members of a bacterial community, we also used phylum-specific primers to resolve the additional diversity of the bacteria in the lakes investigated. We observed similar distribution patterns: no significant decrease in the taxon richness of the bacterioplankton was observed with increasing salinity in the lakes investigated during two consecutive years, despite the use of both phylum-specific primers and general bacterial primers. These results differ strongly from the patterns found for macroorganisms (Fig. 4) and from the results of a number of previous studies of microorganisms (Table S1). We recognize that even the phylum-specific DGGE may still underestimate the taxon richness of the bacteria and that there are non-specific amplicons in PCR analyses. As the resolution of the full microbial diversity of a given habitat remains difficult, it might be important to analyze a set of samples under the same conditions (e.g., the DNA extraction and PCR etc.), as done in this study, to obtain comparable estimates of bacterial diversity. Nevertheless, a recent study also indicated that the salinity-richness patterns deviated from those observed for macroorganisms when high-throughput pyrosequencing of 16S rRNA genes was applied for the estimation of bacterial richness [8].

Furthermore, what underlying processes are responsible for the richness patterns found in the investigated lakes? The pattern of an increase in the number of DGGE bands with salinity up to 1‰ has not been well documented by previous studies. The current physicochemical data set lacks a type of explanatory power for this pattern. In fresh and slightly saline waters, parameters other than salinity might exert relatively more influence on the diversity of the biological community (for instance, macroinvertebrates [4]). The increase in taxon richness may be related to the increase in nutrients in the lakes investigated over the low-salinity range. A marginally significant correlation between the DGGE band numbers and TN at the lower salinity range was observed (Table S4). It has been suggested that increased nutrients and resources may promote bacterial diversity, and that different taxonomic groups of bacteria could exhibit different responses to changes in productivity [44]. The increase in taxon richness could also be a consequence of greater niche availability for both halotolerant species and freshwater species at an intermediate level of salinity. The data presented here are descriptive, and the tests of these hypotheses require further field and empirical approaches.

No obvious decrease in DGGE band numbers at high salinities has been previously observed and microdiversity has been proposed as an explanation [10], [21], [22]. High degrees of microdiversity were also found in other extreme environments, such as the marine Lost City chimneys [46]. This finding is not surprising because DGGE can potentially differentiate single-base-pair substitutions in DNA fragments. Thus, microdiverse groups of bacteria, i.e., groups consisting of several ribotypes differing only slightly in 16S rRNA gene sequences, may appear as two or more bands on DGGE gels [10], [21], [22]. It also might be possible for different sequences to appear in the same band. Nevertheless, our clone library results indicated that based on the rarefaction plots of taxon richness under different sequence-similarity cutoffs, a greater number of closely related sequences occurred at high salinities than at low salinities (Fig. 3).

Our analyses of the two clone libraries representing the bacterial communities from low and high salinities, however, suggest that the recent diversification rate contributes differently to the phylogenetic structures of the high-salinity and freshwater environments. This difference is indicated by the higher gamma value for the community in the hypersaline Chaqia Lake than that for community in the freshwater Kelike Lake. The higher gamma value suggested that the phylogeny's internal nodes in the hypersaline Chaqia Lake are closer to the tips than those in the freshwater Kelike Lake and imply a higher acceleration of the accumulation of lineages [37], [38]. This temporal difference is consistent with the results of the rarefaction plots of Faith's PD for both lakes (Fig. 3), which shows that the additional new sequences added had less effect on the total phylogenetic diversity present in the phylogeny for the hypersaline Chaqia Lake than on that present in the freshwater Kelike Lake, even when different sequence-identity cutoffs were applied. This recent accelerated diversification in the hypersaline Chaqia Lake could have resulted from the unidirectional and predominating influence of the environmental stress, i.e., salinity, accompanied by a high speciation rate in creating closely related species. Such a pattern of phylogenetic clustering near the tree tips may indicate that these closely related taxa share traits important for their persistence in the extreme environment [47]. This pattern is relatively consistent with microdiversity because a recent accelerated diversification will concentrate the nodes towards the tips in a phylogenetic tree [48]. More generally, the results of this study suggested that the difference in the recent accelerated diversification, rather than the microdiversity, resulted in the absence of any obvious pattern of decrease in taxon richness towards higher salinity. However, it should be noted here that only two samples from low and high salinities were analyzed in the phylogenetic context and that replicates were not included. More rigorous experiments are needed to confirm the current findings.

In summary, we observed similar distribution patterns of bacterioplankton taxon richness with phylum-specific primers and general bacterial primers. The richness increased at salinities lower than 1‰, but no additional clear decrease occurred with higher salinities. Although the peak in bacterial richness at lower salinities may be a consequence of higher nutrient levels and niche availability, recent accelerated diversification might have contributed more significantly to the community taxon diversity (microdiversity) of the bacterioplankton at higher salinities. Further studies are needed to determine the generality of the findings of this study in view of the potential biases of the fingerprinting methods and the limited number of lakes included for sequencing. Thorough investigations of the evolutionary processes in extreme environments are necessary for understanding the patterns of genetic diversity in these environments. These investigations will shed light on the global distribution of microbial diversity.

## Supporting Information

Figure S1 **Principal component analysis plot of environmental and spatial factors, indicating that salinity was the primary gradient across all 33 samples.** Concentrations of Na^+^, K^+^, Mg^2+^, SO_4_^2−^ and conductivity significantly positively correlated with salinity (data of all five parameters not shown). The vertical dotted line separates samples with salinities lower/higher than 1‰. The samples taken in 2004 are depicted by filled circles and those from 2005 by empty circles.
(TIF)

Figure S2 **Trends of bacterial taxon richness along the investigated salinity gradient by using the DGGE fingerprinting method with primers (341f/907r) and group-specific primers (SUM, SUM-2, etc).** SUM: total numbers of bands obtained with phylogenetic primers. SUM-2: the SUM bands number without those of primer sets 2. The number 2–8 indicated the primer sets (Table S2). All data were analyzed with LOWESS regression (span 2/3; degree 1). For a clear view, the original data points were not shown, but available when requested.
(TIF)

Table S1 **Effect of salinity on prokaryotic taxonomic richness in various aquatic ecosystems, except for estuary and fast running environments** [**6]**, [7], [9], [10], [21], [22], [53], [54], [55], [56], [57], [58], [59], [60], [61****] **.**
(DOC)

Table S2 **Brief descriptions of the lakes investigated in 2005, sorted by salinity.**
(DOC)

Table S3 **Set of PCR primers used for DGGE fingerprinting and clone library construction of different phylogenetic groups of bacteria** [**29]**, [30], [31], [62], [63], [64], [65], [66], [67], [68], [69****] **.**
(DOC)

Table S4 **Spearman's rank correlation coefficient (rho), with a two-tailed significance, between richness and environmental variables for all samples of the year 2005.**
(DOC)

Text S1 **References for the Supplementary Material.**
(DOC)
