# Supplementary figures and images for: Do Patterns of Bacterial Diversity along Salinity Gradients Differ from Those Observed for Macroorganisms?
Source: PLoS One. 2011 Nov 18;6(11):e27597. doi: 10.1371/journal.pone.0027597 (PMC3220692; doi:10.1371/journal.pone.0027597)

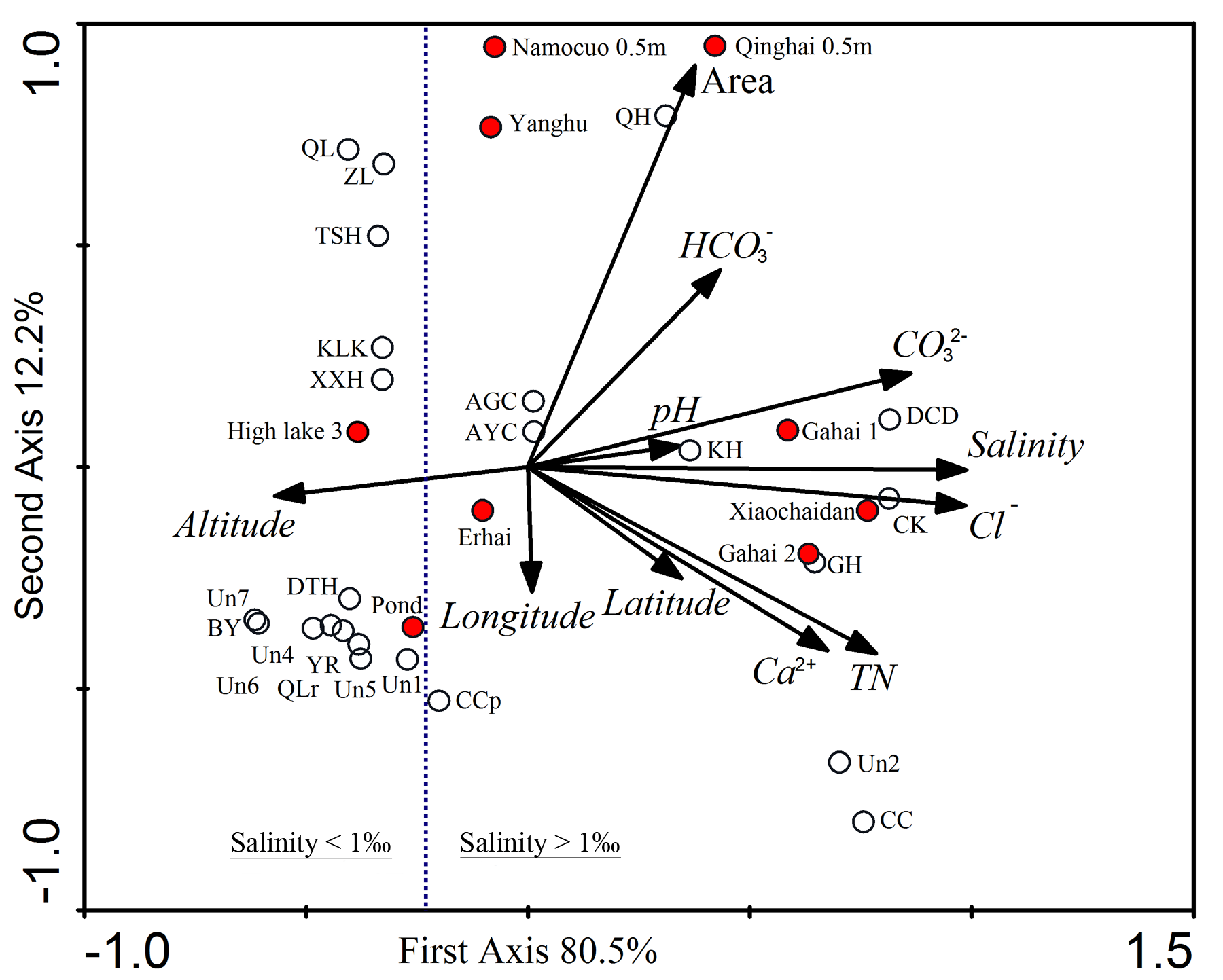

Supplement: Figure S1 — Principal component analysis plot of environmental and spatial factors, indicating that salinity was the primary gradient across all 33 samples. Concentrations of Na+, K+, Mg2+, SO4 2− and conductivity significantly positively correlated with salinity (data of all five parameters not shown). The vertical dotted line separates samples with salinities lower/higher than 1‰. The samples taken in 2004 are depicted by filled circles and those from 2005 by empty circles. (TIF) [file pone.0027597.s001.tif]

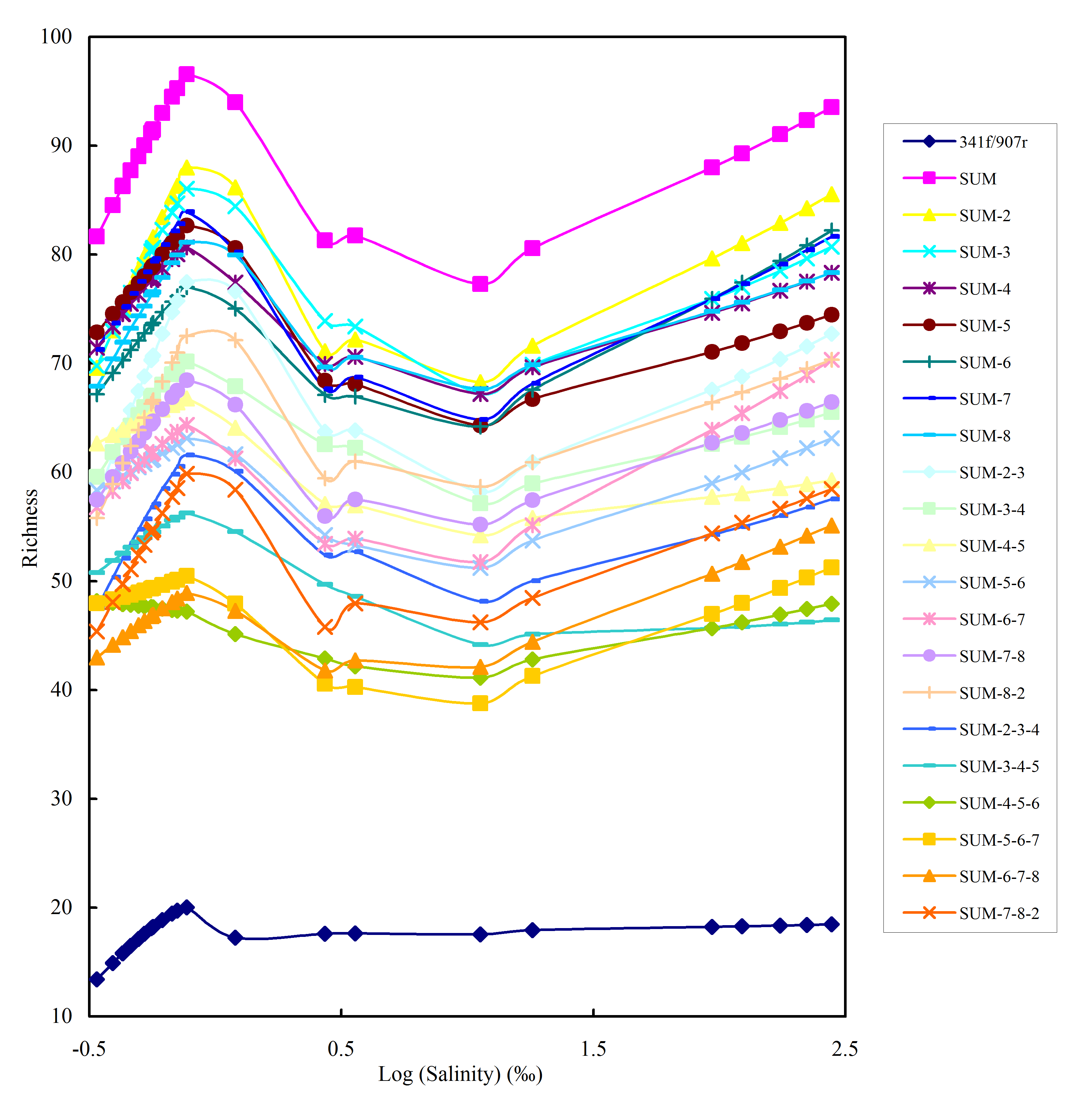

Supplement: Figure S2 — Trends of bacterial taxon richness along the investigated salinity gradient by using the DGGE fingerprinting method with primers (341f/907r) and group-specific primers (SUM, SUM-2, etc). SUM: total numbers of bands obtained with phylogenetic primers. SUM-2: the SUM bands number without those of primer sets 2. The number 2–8 indicated the primer sets (Table S2). All data were analyzed with LOWESS regression (span 2/3; degree 1). For a clear view, the original data points were not shown, but available when requested. (TIF) [file pone.0027597.s002.tif]
