# Supplementary material for: Do Patterns of Bacterial Diversity along Salinity Gradients Differ from Those Observed for Macroorganisms?
Source: PLoS One. 2011 Nov 18;6(11):e27597. doi: 10.1371/journal.pone.0027597 (PMC3220692; doi:10.1371/journal.pone.0027597)
Supplement: Table S1 — Effect of salinity on prokaryotic taxonomic richness in various aquatic ecosystems, except for estuary and fast running environments [6] , [7], [9], [10], [21], [22], [53], [54], [55], [56], [57], [58], [59], [60], [61] . (DOC) [file pone.0027597.s003.doc]

| Salinity (‰) | | Sample No. | Richness along Salinity | Targets | Methods | Environments | Location | Reference |
| --- | --- | --- | --- | --- | --- | --- | --- | --- |
| Lowest | Highest |
| ~100 | ~600 | 5 | Decrease | Bacteria | Enrichment cultures | multi-pond salterns, water | Spain | [9] |
| 64 | 308 | 5 | Decrease | Bacteria | RFLP | Coastal solar salterns, water | Spain | [53] |
| 40 | 370 | 8 | Decrease | Bacteria | DGGE, Clone library | Coastal solar salterns, water | Spain | [10] |
| 1890^$^ | 7550^$^ | 20 | Decrease | Cyanobacteria | DGGE | Rice field, soil | India | [54] |
| 158^$^ | 52900^$^ | 3 | Decrease | Cyanobacteria | Morphology, Clone library | Meltwater ponds, water | Antarctica | [55] |
| 7 | 72 | 4 | Decrease | Bacteria | Clone library | Sludge samples | France | [56] |
| 40 | 370 | 7 | No Decrease | Bacteria/Archaea | DGGE, t-RFLP, RISA | Coastal solar salterns, water | Spain | [21] |
| 0.2 | 223 | 16 | No Decrease | Bacteria | DGGE, RLB | Tibetan lakes, water | China | [22] |
| 60 | 200 | 4 | No Decrease | Bacteria | DGGE | Soda lakes, sediments | Russia | [57] |
| 28 | 37.9 | >100 | Increase, but weak | Bacteria | ARISA | Surface marine water | Global Ocean | [58] |
| 40 | 370 | 8 | Increase/Decrease | Virioplankton | DGGE | Solar saltern ponds | Spain | [59] |
| 220 | 370 | 3 | Increase/Decrease | Bacteria | Reassociation, DGGE, t-RFLP | Coastal solar salterns, water | Spain | [7] |
| <25 | >150 | 20 | Increase/Decrease | Bacteria | Flow cytometry | Coastal lagoon, water | Australia | [60] |
| 30-70 | 120-160 | 2 | Increase | Bacteria | Clone library | Industrial wastewater | Israel | [6] |
| 65 | 200 | 3 | Decrease/increase | Bacteria | Clone library | Intertidal Cyanobacterial mats | Germany | [61] |
| 0.3 | 279.2 | 24 | Increase/No Decrease | Bacteria | DGGE, Clone library | Tibetan lakes, water | China | This study |

^$^, Electrical conductivity, μS cm^-1^; DGGE, denaturing gradient gel electrophoresis; t-RFLP, terminal-restriction fragments length polymorphism; RISA, ribosomal internal spacer analysis; ARISA, amplified ribosomal intergenic spacer analysis; Morphology, Morphological identification; Reassociation, thermal melting and reassociation of community DNA; RLB, reverse line blot hybridization.
