# Supplementary material for: Do Patterns of Bacterial Diversity along Salinity Gradients Differ from Those Observed for Macroorganisms?
Source: PLoS One. 2011 Nov 18;6(11):e27597. doi: 10.1371/journal.pone.0027597 (PMC3220692; doi:10.1371/journal.pone.0027597)
Supplement: Table S2 — Brief descriptions of the lakes investigated in 2005, sorted by salinity. (DOC) [file pone.0027597.s004.doc]

**Table S2 -** Brief descriptions of the lakes investigated in 2005, sorted by salinity. More details of the lakes investigated in 2004 are available in reference (Wu et al., 2006).

| Lakes | Brief Names | Sampling time | Sampling depth  (m) | Maximum depth  (m) | longitude  (E) | Latitude  (N) | Altitude  (m) | Area  (km^2^) | Water temperature (^o^C) | TP  (mg/L) | TN  (mg/L) | pH | Salinity^a^  (‰) |
| --- | --- | --- | --- | --- | --- | --- | --- | --- | --- | --- | --- | --- | --- |
| Unnamed lake-7 | Un7 | 2005-7-15 | 0.5 | 2.6 | 97.466 | 34.104 | 4619 | 0.02 | 15.0 | n.d. | 1.500 | 8.47 | 0.34 |
| Bayan | BY | 2005-7-15 | 0.5 | 1.8 | 98.004 | 34.333 | 4220 | 0.01 | 11.0 | n.d. | 1.594 | 8.28 | 0.39 |
| Oulin Lake | QL | 2005-7-10 | 0.5 | 21.0 | 97.426 | 35.033 | 4279 | 610.7 | 9.5 | n.d. | 1.384 | 8.74 | 0.43 |
| Unnamed lake-6 | Un6 | 2005-7-15 | 0.5 | 0.8 | 97.515 | 34.132 | 4606 | 0.03 | 11.0 | n.d. | 2.408 | 8.95 | 0.43 |
| Oulin Reservoir | QLr | 2005-7-10 | 0.5 | 4.8 | 97.501 | 35.061 | 4278 | 0.13 | 11.5 | n.d. | 1.347 | 8.57 | 0.46 |
| Unnamed lake-5 | Un5 | 2005-7-15 | 0.5 | 0.6 | 98.004 | 34.333 | 4220 | 0.02 | 11.0 | n.d. | 1.923 | 9.19 | 0.50 |
| Unnamed lake-4 | Un4 | 2005-7-11 | 0.5 | 1.0 | 98.082 | 34.476 | 4256 | 0.001 | 9.5 | n.d. | 2.026 | 9.18 | 0.52 |
| Daotanhe | DTH | 2005-7-18 | 0.5 | 1.1 | 100.740 | 36.577 | 3210 | 0.5 | 15.6 | 1.016 | 1.974 | 9.55 | 0.56 |
| Yellow River | YR | 2005-7-10 | 0.5 | 1.7 | 98.113 | 34.520 | 4228 | n.d. | 9.8 | n.d. | 1.351 | 8.55 | 0.56 |
| Tuisuhu | TSH | 2005-7-14 | 0.5 | 20 | 98.306 | 35.154 | 4082 | 200 | 12.5 | n.d. | 1.252 | 8.83 | 0.57 |
| Zhalin Lake | ZL | 2005-7-08 | 0.5 | 7.5 | 97.213 | 34.514 | 4298 | 526 | 9.5 | n.d. | 1.352 | 8.42 | 0.62 |
| Xinxinhai | XXH | 2005-7-07 | 0.5 | ND | 98.075 | 34.505 | 4219 | 29.3 | 13.0 | n.d. | 1.723 | 8.47 | 0.67 |
| Kelike | KLK | 2005-7-21 | 0.5 | 2.5 | 96.964 | 37.380 | 2797 | 56.7 | 19.8 | n.d. | 1.629 | 8.99 | 0.71 |
| Unnamed lake-1 | Un1 | 2005-7-10 | 0.4 | 0.6 | ND | ND | 4268 | 0.002 | 7.8 | n.d. | 1.603 | 10.3 | 1.00 |
| Chacuo Pond | CCp | 2005-7-10 | 0.3 | 0.4 | 97.372 | 34.582 | 4293 | 5 (m^2^) | 10.6 | 1.025 | 3.208 | 8.46 | 1.20 |
| Ayongcuo | AYC | 2005-7-16 | 0.3 | 0.4 | 98.165 | 34.474 | 4202 | 22.7 | 12.5 | 1.036 | 6.144 | 9.34 | 2.72 |
| Agecuo | AGC | 2005-7-16 | 0.5 | 9.0 | 98.102 | 34.471 | 4216 | 37.6 | 9.2 | n.d. | 1.863 | 9.05 | 3.59 |
| Qinghai (near river) | QH | 2005-7-18 | 0.5 | 23 | 100.680 | 36.590 | 3203 | 4340 | 14.5 | 1.005 | 1.926 | 9.16 | 11.28 |
| Kuhai | KH | 2005-7-14 | 0.5 | 14 | 99.115 | 35.192 | 4130 | 44.4 | 14.0 | 1.016 | 2.771 | 8.80 | 18.13 |
| Gahai2 | GH | 2005-7-21 | 0.5 | 0.8 | 97.550 | 37.150 | 2855 | 32 | 18.7 | 1.075 | 3.942 | 8.38 | 93.66 |
| Unnamed lake-2 | Un2 | 2005-7-07 | 0.5 | ND | 97.224 | 34.495 | 4290 | 2 | 15.0 | 1.065 | 8.530 | 9.43 | 122.91 |
| Chacuo | CC | 2005-7-10 | 0.4 | 0.4 | 97.379 | 34.581 | 4283 | 1.2 | 13.0 | 1.336 | 13.143 | 9.24 | 174.23 |
| Dachaidan | DCD | 2005-7-21 | 0.5 | 0.5 | 95.080 | 37.830 | 2790 | 200 | 16.7 | 1.032 | 7.263 | 9.76 | 222.62 |
| Chaqia | CQ | 2005-7-22 | 0.5 | 0.6 | 99.079 | 36.750 | 3064 | 105 | 16.8 | 1.045 | 9.307 | 9.71 | 279.17 |

ND: not determined; n.d.: not detectable; ^a^ Sum of the ions potassium, sodium, calcium, magnesium, chloride, sulfate, carbonate and bicarbonate.
