# Supplementary material for: Do Patterns of Bacterial Diversity along Salinity Gradients Differ from Those Observed for Macroorganisms?
Source: PLoS One. 2011 Nov 18;6(11):e27597. doi: 10.1371/journal.pone.0027597 (PMC3220692; doi:10.1371/journal.pone.0027597)
Supplement: Table S3 — Set of PCR primers used for DGGE fingerprinting and clone library construction of different phylogenetic groups of bacteria [29] , [30], [31], [62], [63], [64], [65], [66], [67], [68], [69] . (DOC) [file pone.0027597.s005.doc]

| Group | No. | Annealing in PCR ^$^ | Nested PCR | DGGE | Clones | Primers ^a^ | Sequences (5' 🡪 3') | References |
| --- | --- | --- | --- | --- | --- | --- | --- | --- |
| All groups |  | 58^o^C 30s 30(Clone) /25 (nested-PCR) cycles |  |  | + | 8f | AGAGTTTGATCMTGGCTCAG | [30] |
|  |  |  |  |  |  | 1492r | TACGGYTACCTTGTTACGACTT | [30] |
| All groups | 1 | 65^o^C 30s 10cycles ^g^  55^o^C 30s 15cycles |  | + |  | 341f | CTCCTACGGGAGGCAGCAG ^b^ | [31] |
|  |  |  |  |  |  | 907r | CCGTCAATTCCTTTGAGTTT | [31] |
| *Actinobacteria* | 2 | 62^o^C 45s 10cycles  58^o^C 45s 15cycles | + | + | + | 517f | GTGCCAGCAGCCGCGG ^b^ | [30,29] |
|  |  |  |  |  |  | AB1165r | ACCTTCCTCCGAGTTRAC | [62] |
| *Bacteroidetes* | 3 | 60^o^C 30s 10cycles  55^o^C 30s 15cycles | + | + | + | CFB319f | CGTACTGAGACACGGACCA ^d^ | [63,64] |
|  |  |  |  |  |  | 907r | CCGTCAATTCCTTTGAGTTT | [65] |
| *Cyanobacteria* | 4 | 60^o^C 60s 25cycles | + | + | + | CYA359f | GGGGAATYTTCCGCAATGGG ^e^ | [66] |
|  |  |  |  |  |  | CYA781r(a) ^f^ | GACTACTGGGGTATCTAATCCCATT | [66] |
|  |  |  |  |  |  | CYA781r(b) ^f^ | GACTACAGGGGTATCTAATCCCTTT | [66] |
| *Firmicutes* | 5 | 56^o^C 45s 10cycles  51^o^C 45s 15cycles | + | + | + | LGC354f | GCAGTAGGGAATCTTCSR ^b^ | [29,67] |
|  |  |  |  |  |  | 907r | CCGTCAATTCCTTTGAGTTT | [31] |
| *Planctomyces* | 6 | 68^o^C 45s 10cycles  65^o^C 45s 15cycles | + | + | + | 8f | AGAGTTTGATCMTGGCTCAG | [30] |
|  |  |  |  |  |  | PLA886r | GCCTTGCGACCATACTCCC | [29,67] |
| *α-Proteobacteria* | 7 | 66^o^C 30s 10cycles  61^o^C 30s 15cycles | + | + | + | 517f | GTGCCAGCAGCCGCGG ^b^ | [30,29] |
|  |  |  |  |  |  | Alf968r | GGTAAGGTTCTGCGCGTT | [29] |
| *β-Proteobacteria* | 8 | 58^o^C 30s 10cycles  54^o^C 30s 15cycles | + | + | + | Beta680f | CRCGTGTAGCAGTGA | [29,68] |
|  |  |  |  |  |  | 1055r | AGCTGACGACAGCCAT ^b^ | [69] |

^$^ A typical PCR reaction was carried out in 25-μl volumes and contained 0.2 mM dNTP, 1.5 mM MgCl_2_, 0.2 mM of each primer, 2.5 μl buffer (10×TE Buffer, Takara, China), 0.8 U Taq polymerase (Takara, Dalian, China) and 10 ng of template DNA. Each protocol included a single 4-min cycle at 96°C at the beginning, 30 s at 94°C (denaturation), 1 min at 72°C (extension) and terminated with a 10-min cycle at 72°C. ^a^ f, forward; r, reverse; GC clamp is attached to the 5' end of the forward primers for DGGE. ^b^ GC clamp: 5’-CGCCCGCCGCGCCCCGCGCCCGGCCCGCCGCCCCCGCCCC-3’.

^d^ GC clamp: 5’-CGCCCGCCGCCGCCCCGCGCCCGGCCCGCCGCCCCCGCCC-3’. ^e^ GC clamp: 5’-CGCCCGCCGCGCCCCGCGCCGGTCCCGCCGCCCCCGCCCG.-3’

^f^ Reverse primer CYA781R was an equal molar mixture of CYA781R(a) and CYA781R(b). ^g^ The annealing temperature of the reaction is decreased 1 °C every second cycle.

95°C for 30 s, 58°C for 30 s and 72°C for 2 min.
