# Supplementary material for: Do Patterns of Bacterial Diversity along Salinity Gradients Differ from Those Observed for Macroorganisms?
Source: PLoS One. 2011 Nov 18;6(11):e27597. doi: 10.1371/journal.pone.0027597 (PMC3220692; doi:10.1371/journal.pone.0027597)
Supplement: Table S4 — Spearman's rank correlation coefficient (rho), with a two-tailed significance, between richness and environmental variables for all samples of the year 2005. (DOC) [file pone.0027597.s006.doc]

**Table S4** - Spearman’s rank correlation coefficient (rho), with a two-tailed significance, between richness and environmental variables for all samples of the year 2005.

| Primer pairs | Salinity range |  | Salinity | pH | Conductivity | TN | TP | HCO_3_^-^ | CO_3_^2-^ | Cl^-^ | SO_4_^2-^ | K^+^ | Na^+^ | Ca^2+^ | Mg^2+^ |
| --- | --- | --- | --- | --- | --- | --- | --- | --- | --- | --- | --- | --- | --- | --- | --- |
| 341f/907r | Whole salinity | rho | 0.25 | 0.15 | 0.28 | 0.28 | 0.09 | 0.00 | 0.37 | 0.22 | 0.16 | 0.17 | 0.08 | 0.01 | 0.27 |
|  |  | *P* | 0.25 | 0.48 | 0.19 | 0.18 | 0.66 | 1.00 | 0.08 | 0.30 | 0.45 | 0.43 | 0.72 | 0.95 | 0.20 |
|  | < 1‰ | rho | 0.55 | 0.26 | 0.53 | 0.48 | 0.19 | -0.07 | 0.81 | 0.51 | 0.21 | 0.32 | 0.17 | -0.52 | 0.61 |
|  |  | *P* | 0.05^*^ | 0.38 | 0.06 | 0.10 | 0.53 | 0.82 | 0.00^*^ | 0.08 | 0.49 | 0.28 | 0.57 | 0.07 | 0.03^*^ |
| SUM^$^ | Whole salinity | rho | 0.15 | 0.15 | 0.06 | 0.16 | 0.01 | -0.27 | 0.00 | 0.16 | 0.08 | 0.07 | 0.04 | -0.02 | 0.12 |
|  |  | *P* | 0.49 | 0.48 | 0.76 | 0.47 | 0.95 | 0.20 | 0.99 | 0.45 | 0.70 | 0.74 | 0.85 | 0.94 | 0.57 |
|  | < 1‰ | rho | 0.67 | -0.03 | 0.47 | 0.55 | -0.04 | 0.00 | 0.30 | 0.71 | 0.48 | 0.39 | 0.28 | -0.32 | 0.58 |
|  |  | *P* | 0.01^*^ | 0.93 | 0.11 | 0.05^*^ | 0.90 | 0.99 | 0.32 | 0.01 | 0.10 | 0.18 | 0.35 | 0.28 | 0.04^*^ |

**^$^** SUM: total numbers of bands obtained with phylogenetic primers.^*^ significant*,P* < 0.05.
