# Supplementary material for: Do Patterns of Bacterial Diversity along Salinity Gradients Differ from Those Observed for Macroorganisms?
Source: PLoS One. 2011 Nov 18;6(11):e27597. doi: 10.1371/journal.pone.0027597 (PMC3220692; doi:10.1371/journal.pone.0027597)
Supplement: Text S1 — References for the Supplementary Material. (DOC) [file pone.0027597.s007.doc]

**References of relevance for the presented Supplementary Materials**

1. Lane DJ (1991) 16S/23S rRNA sequencing. In: Stackebrandt E, Goodfellow M, editors. Nucleic acid techniques in bacterial systematics: Wiley, New York. pp. 115-175.

2. Muyzer G, de Waal EC, Uitterlinden AG (1993) Profiling of complex microbial populations by denaturing gradient gel electrophoresis analysis of polymerase chain reaction-amplified genes coding for 16S rRNA. Applied and Environmental Microbiology 59: 695-700.

3. Gich F, Schubert K, Bruns A, Hoffelner H, Overmann J (2005) Specific detection, isolation, and characterization of selected, previously uncultured members of the freshwater bacterioplankton community. Applied and Environmental Microbiology 71: 5908-5919.

4. Ludemann H, Conrad R (2000) Molecular retrieval of large 16S rRNA gene fragments from an Italian rice paddy soil affiliated with the class Actinobacteria. Systematic and Applied Microbiology 23: 582-584.

5. Manz W, Amann R, Ludwig W, Vancanneyt M, Schleifer KH (1996) Application of a suite of 16S rRNA-specific oligonucleotide probes designed to investigate bacteria of the phylum cytophaga-flavobacter-bacteroides in the natural environment. Microbiology 142: 1097-1106.

6. Jaspers E, Nauhaus K, Cypionka H, Overmann J (2001) Multitude and temporal variability of ecological niches as indicated by the diversity of cultivated bacterioplankton. FEMS Microbiology Ecology 36: 153-164.

7. Nübel U, Garcia-Pichel F, Muyzer G (1997) PCR primers to amplify 16S rRNA genes from cyanobacteria. Applied and Environmental Microbiology 63: 3327-3332.

8. Meier H, Amann R, Ludwig W, Schleifer KH (1999) Specific oligonucleotide probes for in situ detection of a major group of gram-positive bacteria with low DNA G + C content. Systematic and Applied Microbiology 22: 186-196.

9. Neef A, Amann R, Schlesner H, Schleifer KH (1998) Monitoring a widespread bacterial group: in situ detection of planctomycetes with 16S rRNA-targeted probes. Microbiology-UK 144: 3257-3266.

10. Overmann J, Coolen MJL, Tuschak C (1999) Specific detection of different phylogenetic groups of chemocline bacteria based on PCR and denaturing gradient gel electrophoresis of 16S rRNA gene fragments. Archives of Microbiology 172: 83-94.

11. Amann RI, Ludwig W, Schleifer KH (1995) Phylogenetic identification and in-situ detection of individual microbial-cells without cultivation. Microbiological Reviews 59: 143-169.
